# Purification and Biochemical Characterization of a New Protease Inhibitor from *Conyza dioscoridis* with Antimicrobial, Antifungal and Cytotoxic Effects

**DOI:** 10.3390/molecules25225452

**Published:** 2020-11-20

**Authors:** Aida Karray, Mona Alonazi, Slim Smaoui, Philippe Michaud, Dina Soliman, Abir Ben Bacha

**Affiliations:** 1 Laboratoire de Biochimie et de Génie Enzymatique des Lipases, ENIS Route de Soukra, Université de Sfax, Sfax 3038, Tunisia; aida.karray@enis.tn; 2 Biochemistry Department, Science College, King Saud University, P.O. Box 22452, Riyadh 11495, Saudi Arabia; moalonzi@ksu.edu.sa; 3 Laboratoire de Microorganismes et de Biomolécules du Center de Biotechnology de Sfax, Route Sidi Mansour km 6 B.P. 117, Sfax 3018, Tunisia; slim.smaoui@yahoo.fr; 4 Institut Pascal, CNRS, SIGMA Clermont, Université Clermont Auvergne, F-63000 Clermont-Ferrand, 63178 Aubière, France; philippe.michaud@uca.fr; 5 Botany and Microbiology Department, Science College, King Saud University, P.O. Box 22452, Riyadh 11495, Saudi Arabia; dsoliman@ksu.edu.sa; 6 Laboratory of Plant Biotechnology Applied to Crop Improvement, Faculty of Science of Sfax, University of Sfax, Sfax 3038, Tunisia

**Keywords:** *Conyza dioscoridis*, protease inhibitor, characterization antimicrobial effect, protein therapeutics

## Abstract

The main objective of the current study was the extraction, purification, and biochemical characterization of a protein protease inhibitor from *Conyza* *dioscoridis*. Antimicrobial potential and cytotoxic effects were also examined. The protease inhibitor was extracted in 0.1 M phosphate buffer (pH 6–7). Then, the protease inhibitor, named PDInhibitor, was purified using ammonium sulfate precipitation followed by filtration through a Sephadex G-50 column and had an apparent molecular weight of 25 kDa. The N-terminal sequence of PDInhibitor showed a high level of identity with those of the Kunitz family. PDInhibitor was found to be active at pH values ranging from 5.0 to 11.0, with maximal activity at pH 9.0. It was also fully active at 50 °C and maintained 90% of its stability at over 55 °C. The thermostability of the PDInhibitor was clearly enhanced by CaCl_2_ and sorbitol, whereas the presence of Ca^2+^ and Zn^2+^ ions, Sodium taurodeoxycholate (NaTDC), Sodium dodecyl sulfate (SDS), Dithiothreitol (DTT), and β-ME dramatically improved the inhibitory activity. A remarkable affinity of the protease inhibitor with available important therapeutic proteases (elastase and trypsin) was observed. PDInhibitor also acted as a potent inhibitor of commercial proteases from *Aspergillus oryzae* and of Proteinase K. The inhibitor displayed potent antimicrobial activity against gram+ and gram- bacteria and against fungal strains. Interestingly, PDInhibitor affected several human cancer cell lines, namely HCT-116, MDA-MB-231, and Lovo. Thus, it can be considered a potentially powerful therapeutic agent.

## 1. Introduction

Proteases represent up to 2% of the total human genome, with 588 identified proteases [1,2]. According to the protease database MEROPS, they are classified based on catalytic mechanisms into seven classes: serine, aspartic, cysteine, threonine, glutamic, asparagine, and metalloproteases [3,4]. The largest known family of proteases is serine proteases [5]. In general, proteases play key roles in different physiological processes in all living organisms, including replication, transcription, cell proliferation, cell migration, tissue morphogenesis, release of hormones and pharmacologically active peptides, apoptosis, and wound healing [1,6,7].

However, dysregulation of proteases is implicated in the pathogenic process of several human diseases; therefore, protease inhibitors (PIs) are considered potentially powerful therapeutic agents against several diseases [8,9]. Among several protease inhibitors, serine protease inhibitors have received significant interest in various applications, mainly focused on therapeutics. According to the classification system proposed by Fasalli et al. [10], PIs have been grouped into 85 families and 38 clans [11]. One of the most common sources of serine PIs is plants, and those serine PIs are classified as Bowman-Birk, Kunitz, Potato I, Potato II, Serpine, Cereal, Rapeseed, Mustard, and Squash serine PIs [11,12]. Currently, well-established PIs, such as angiotensin-converting enzyme, renin, and thrombin inhibitors, are used for treatment of cardiovascular disorders, primary hypertension, and congestive heart failure. In addition, HIV protease inhibitors, proteasome inhibitors for myeloma, and dipeptidyl peptidase IV inhibitors for type II diabetes are used clinically [6,13]. However, discovering new specific and selective PIs has emerged as an essential need in drug development.

*Conyza dioscoridis* (L.) Desf. is a richly branched hairy shrub belonging to the Asteraceae family and is found in the Middle East and tropical African countries. In folk medicine, different parts of *C. disocoridis* are used as a popular remedy for treatment of epilepsy in children, in addition to relieving rheumatic pains, flatulence, colic, ulcers, and colds [14,15,16]. Recent chemical studies have reported that *C. dioscoridis* is a rich source of different potentially bioactive compounds, such as phenolic acids, flavonoids, sulfated flavonoids, steroids, and sesquiterpenoids [17]. A few studies have demonstrated the biological activity of extracts of different parts of C. *disocoridis*, such as anti-diarrheal, antimicrobial, anti-inflammatory [15], hypoglycemic [18], and anti-ulcerative colitis activities [19,20]. This study aimed to evaluate the inhibitory activity of PIs isolated from *C. dioscoridis* against some commercially and therapeutically important proteases, in addition to their antimicrobial and cytotoxicity potential.

## 2. Results

### 2.1. Extraction of Protease Inhibitor from C. dioscoridis, Solvent Optimization

Among a range of extraction solvents tested for improvement of protease inhibitor activity extracted from C. dioscoridis, the resulting soluble crude extracts prepared in 0.1 M phosphate buffer showed maximum protease inhibitor activity (83.3% ± 3.05) followed by those prepared in 15% sodium chloride (66.3% ± 4.1) (Table 1). Much lower trypsin inhibitor activity was found with extracts prepared in distilled water, HCl and NaOH, with inhibition rates of 47.3% ± 2.5, 35% ± 3.6, and 18.6% ± 3.5, respectively.

### 2.2. Purification of Protease Inhibitor from C. dioscoridis (PDInhibitor)

PDInhibitor was purified successively from soluble crude extracts prepared in phosphate buffer by the procedure described in the Materials and Methods. The purification flow sheet is summarized in Table 2.

**Figure 1:**
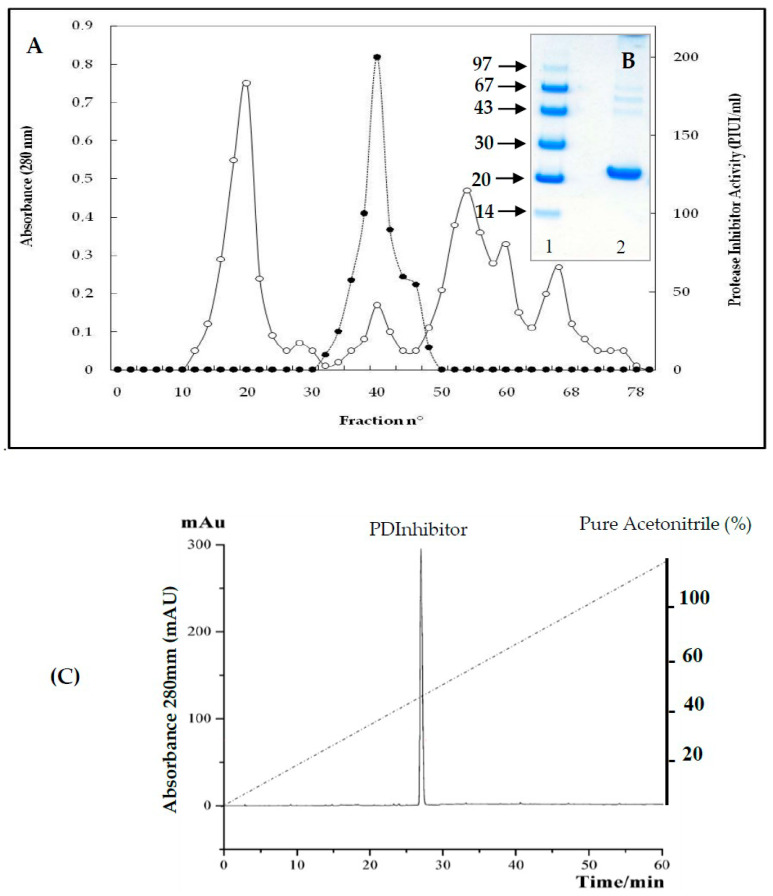
Purification of PDInhibitor. (**A**) Gel filtration chromatography using a Sephadex G-50 column. A G-50 column (2 × 100 cm) was equilibrated with 0.1 Μ Tris-HCL buffer (pH 8) containing 0.2 Μ NaCl. The column was eluted at 4 °C with the same solution at a flow rate of 30 mL/hand, and 4.0 mL fractions were collected. (**B**) SDS-PAGE for Conyza dioscoridis protease inhibitor. 1: molecular markers (14 kD, 20 kD, 30 kD, 43 kD, 67 kD and 97 kD); 2: Sephadex G-50 PDInhibitor. (**C**) A reversed-phase analytical HPLC on C-8 column. A reverse phase RP-HPLC eurospher 100, C-8 column (250 × 4.6 mm), was equilibrated with 0.1% TFA in water. Protein elution was performed with an acetonitrile linear gradient (0–100%) at a flow rate of 1 mL/min over 60 min. 20µL of PDInhibitor (1 mg/mL) was used.

Figure 1A shows the elution profile after gel filtration through a Sephadex G-50 column (2 × 100 cm) equilibrated with 0.1 Μ Tris HCL buffer, pH 8, containing 0.2 Μ NaCl. The column was eluted at 4 °C with the same solution at a flow rate of 30 mL/hand, and 4.0 mL fractions were collected. The fractions exhibiting PDInhibitor activity emerged in a single peak between 1.5 and 2 void volumes (Figure 1A). After the three purification steps, PDInhibitor was purified 76.7-fold, with a recovery of 26%. It showed a specific activity of 1189.1 PIU/mg. The active fractions exhibiting protease inhibitor activity were pooled and analyzed via SDS-PAGE under reducing conditions (Figure 1B). The figure shows that PDInhibitor is homogenously pure and has an apparent molecular mass of approximately 25 kDa. A reversed-phase analytical HPLC eurospher 100, C-8 column (250 × 4.6 mm) showed homogeneity of the identified inhibitor. The column was equilibrated with 0.1% TFA in water and protein elution was performed with an acetonitrile linear gradient (0–100%) (Figure 1C).

**Figure 2:**
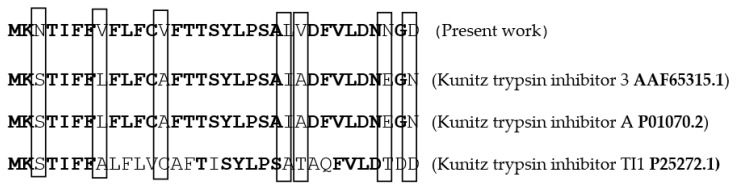
N-terminal sequence analysis of PDInhibitor from C. dioscoridis: Identical amino acids are in bold, and different amino acids are in boxes.

The NH2-terminal sequence (the first 33 residues) of the purified PDInhibitor was determined (MKNTIFFVFLFCVFTTSYLPSALVDFVLDNNGD). Figure 2 shows the alignment of the N-terminal sequence of the protease inhibitor from *C. dioscoridis* (present work) with several other related sequences of Kunitz-type protease inhibitors. The sequence showed a high level of identity with that of Kunitz trypsin inhibitor 3 (79%) [22], Kunitz trypsin inhibitor A (79%) [23], and Kunitz trypsin inhibitor TI1 (65%) [23], with accession numbers AAF65315.1, P01070.2, and P25272.1, respectively.

### 2.3. Biochemical Characterization of the Purified PDInhibitor

#### 2.3.1. Effect of pH and Temperature on PDInhibitor Activity and Stability

**Figure 3:**
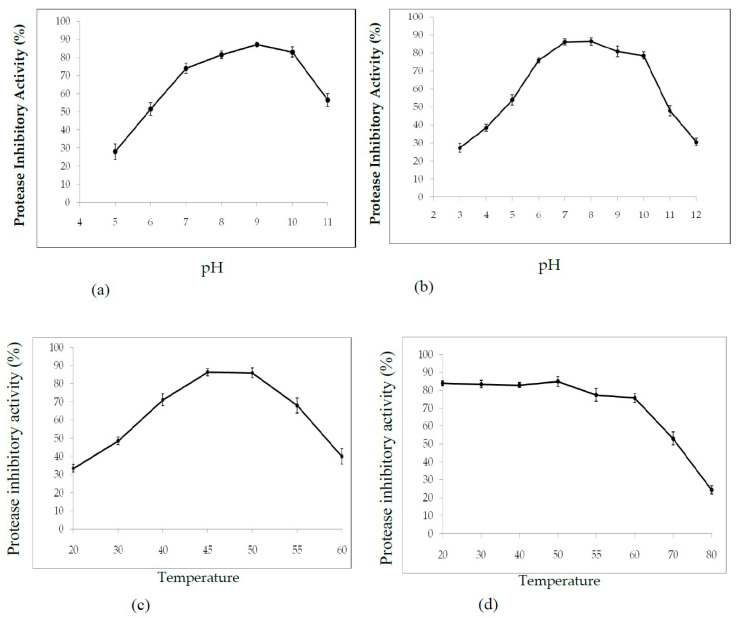
Effect of pH and temperature on PDInhibitor activity: Effect of pH on PDInhibitor activity (**a**) and stability (**b**). Inhibitor activity was assayed against trypsin at various pH values. For stability studies, the protease inhibitor was incubated in medium with different pH values for 12 h and assayed for residual inhibitor activity under standard conditions. Effect of temperature on *PDInhibitor* activity (**c**) and stability (**d**). Inhibitor activity was assayed against trypsin at various temperatures. For stability studies, the protease inhibitor was incubated at different temperatures, drawn at various time intervals and assayed for residual inhibitor activity under optimal pH and temperature conditions. The data shown are the mean ± SD (*n* = 3).

The effects of pH on PDInhibitor activity and stability were examined. As shown in Figure 3, the maximum protease inhibitor activity was obtained at pH 9 (87% ± 1.1% inhibition). Nearly the same activity levels were observed at pH 8 and 10, with 81% and 83% activity, respectively (Figure 3a). The observed decrease in inhibitory activity under highly acidic conditions (pH 2.0–4.0) indicated denaturation of the protease inhibitor to reach 28% activity at pH 5. Interestingly, we noted that the purified protease inhibitor was highly stable at pH 7–10 (Figure 3b). Moreover, the inhibitor maintained more than 48% of its activity at pH 11 for 12 h (Figure 3b).

The influence of temperature on the activity and stability of the purified protease inhibitor was assessed using a standard assay. The results shown in Figure 3c verify that purified PDInhibitor maintained activity at temperatures ranging from 20 to 60 °C, with maximal protease inhibition activity of 86% ± 2 at 50 °C. Interestingly, the protease inhibitor activity was preserved over a wide range of temperatures, even under extreme conditions, with more than 50% of the PDInhibitor activity being conserved at 70 °C (Figure 3d).

#### 2.3.2. Influence of Stabilizers on PDInhibitor Thermo-Stability

**Figure 4:**
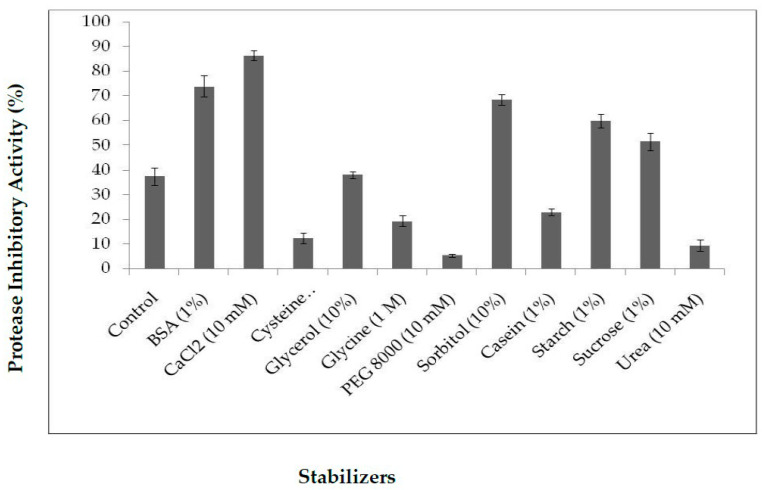
Effect of stabilizers on PDInhibitor activity at 70 °C. The inhibitor activity was determined under standard assay conditions (pH 7 and 37 °C) after 4 h of incubation of the inhibitor protein with various thermo stabilizers. The data are the means of triplicate determinations ± SD (*n* = 3).

The thermal stability of protease inhibitors is essential for biotechnological applications, and it is thought to be affected by stabilizer additives. Thus, thermal stability at 70 °C was studied in the presence of several additives, such as BSA, CaCl_2_, urea, glycerol, glycine, PEG 8000, sorbitol, and casein. Inhibitor activity in the absence of stabilizer was used as a control. Purified PDInhibitor showed thermal stability and inhibitory activity in the presence of all the stabilizers except urea, PEG 8000, and cysteine. (Figure 4). Therefore, maximum stability was promoted as follows: 10 mM CaCl_2_ (86% inhibition) > BSA (70%) > sorbitol (68%) > starch (60%) > sucrose (50%). Glycerol and casein supported moderate thermal stability, with values of 38% and 28%, respectively.

#### 2.3.3. Effect of Various Metal Ions on Protease Inhibitor Activity

**Figure 5:**
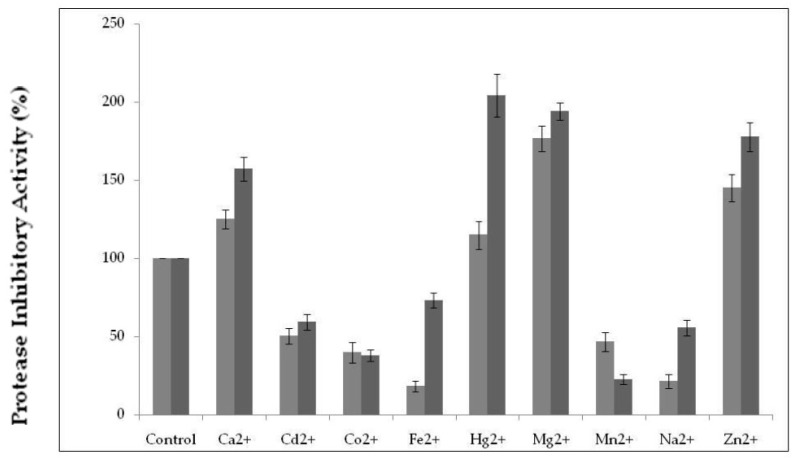
Effects of metal ions at different concentrations on PDInhibitor activity. The protease inhibitor assay was performed at 37 °C and pH 7. The control represents 100% of the protease inhibitor activity under the same conditions in the absence of any metal. The data shown are the mean ± SD (*n* = 3).

Monovalent and divalent metal ions play a crucial role in maintaining the structural integrity of the secondary and tertiary structure of cysteine protease inhibitors. Protease inhibitor activity was studied in the presence of 1 and 10 mM concentrations of metal ions. Inhibitory activity measured in the absence of metal ions was considered 100% and taken as a control. Figure 5 shows that the divalent metal ions Ca^2+^ and Hg^2+^ at 1 mM enhanced residual protease inhibitor activity only up to a marginal level (115 and 125%, respectively). However, the presence of Mg^2+^ and Zn^2+^ at 10 mM improved PDInhibitor inhibitory activity by up to 204 and 177%, respectively, compared to the residual activity of the control. In contrast, the divalent ions Cd^2+^, Co^2+^ and Mn^2+^ at 10 mM reduced the inhibitor activity by up to 50%, 38%, and 22%, respectively.

### 2.4. Effect of various Reducing/Oxidizing Agents and Surfactants on PDInhibitor Activity

Among the ionic and nonionic surfactants tested, only SDS and NaTDC activated the purified protease inhibitor by 141% and 193%, respectively. However, the inhibitor protein maintained more than 80% of its initial activity in the presence of Tween 20 and 64% in the presence of Tween 80 and Tween-X100 compared to the control. (Table 3). Oxidizing agents, such as H_2_O_2,_ NaOCl and DMSO, induced a decrease in PDInhibitor activity with an increase in the concentration of the oxidizing agents. DMSO was found to moderately preserve stability at 1% and 2% DMSO, with 89% and 53% inhibitory activity, respectively. The presence of 1% and 2% H_2_O_2_ did not strongly affect the inhibitor activity, and we noted 58% and 40% inhibitor activity, respectively. Beyond 2%, a significant decrease in the inhibitory activity to 18% and 8% was noted at H_2_O_2_ (*v*/*v*) concentrations of 3% and 4%, respectively. Furthermore, the effect of reducing agents (0.2–1%) on the activity of the protease inhibitor was studied, and the results are presented in Table 2. Both DTT and βME were found to improve the activity of the protease inhibitor. Indeed, the inhibitory activity was strongly increased from 7% to 60% along with a corresponding increase in the concentration of DTT from 0.2% to 1%. Nearly the same behavior was observed in the presence of the reducing agent βME at 0.2% and 1% concentrations, with an increase in the inhibitory activity of 3% and 67%, respectively.

### 2.5. Effect of PDInhibitor on Available Important Therapeutic and Commercial Proteases

**Figure 6:**
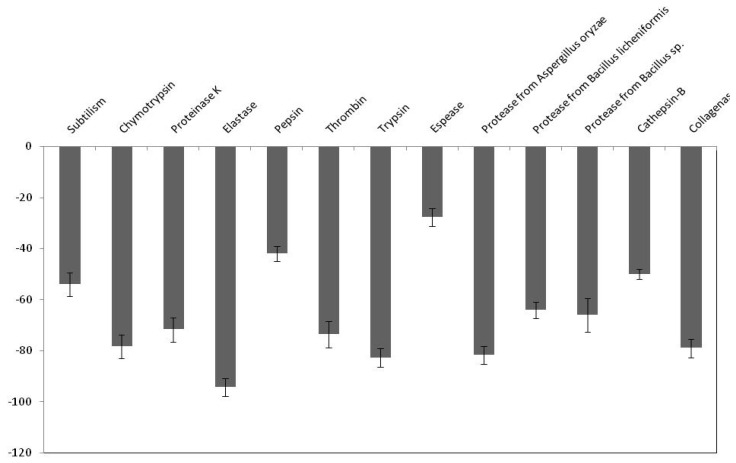
Inhibitory activity of PDInhibitor toward several proteases with pharmaceutical and commercial importance. Data are the means of triplicate determinations ± SD.

We first measured the effect of the purified protease inhibitor from C. dioscoridis on several important therapeutic proteases, such as cathepsin, elastase, and thrombin. It was noted from the results presented in Figure 6, that purified PDInhibitor seems to possess the highest affinity toward elastase (94% inhibitory activity), followed by trypsin and chymotrypsin (82% and 78%, respectively). Overall, the same inhibitory effect was observed with chymotrypsin, collagenase, and thrombin (79%, 78% and 73%, respectively). Moreover, the protease inhibitor exhibited much less affinity toward pepsin and espase (42% and 27%, respectively). Next, the effect of PDInhibitor on six industrially important proteases was investigated. Visibly, the data presented in Figure 6 show that the commercial protease obtained from Aspergillus oryzae (81% inhibition) was dramatically inhibited by the C. disocoridis protease inhibitor. In addition, proteinase K and proteases from Bacillus licheniformis and Bacillus sp. possess comparative inhibitory activity when incubated with PDInhibitor (71%, 66% and 64%, respectively). The lowest inhibitory effect was found against esperase (26%).

### 2.6. Antimicrobial Activity of the Purified PDInhibitor

**Figure 7:**
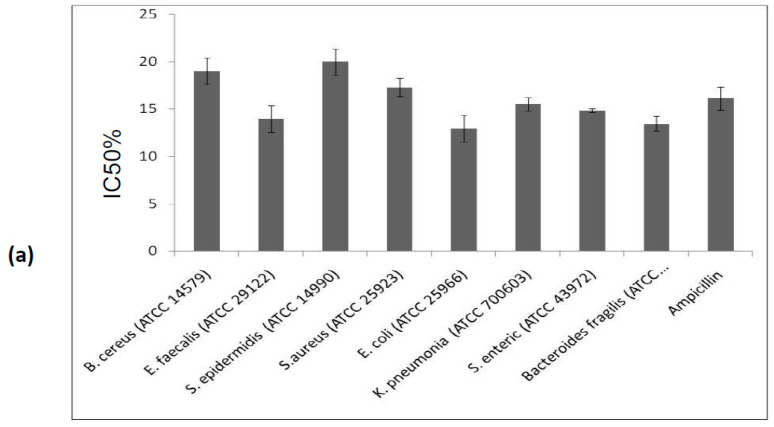

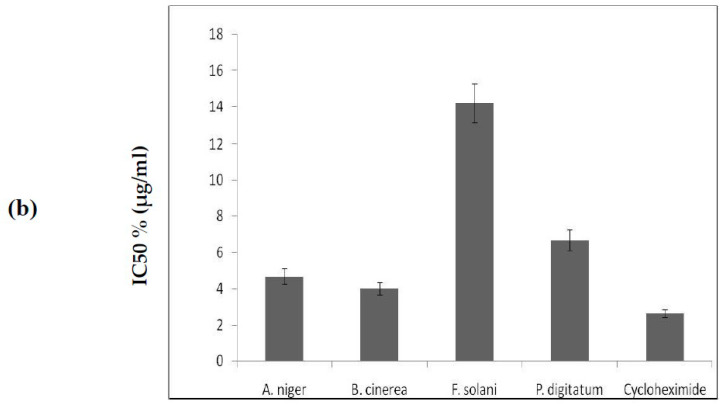
(**a**): Antibacterial properties of the purified protease inhibitor PDInhibitor. The antibacterial properties of PDInhibitor were evaluated against several gram-positive and gram-negative bacteria. The bactericidal effect was assessed by measuring the protease inhibitor concentration necessary to kill 50% of the initial inoculum (IC50%), which was deduced from curves obtained from three independent experiments. Ampicillin was used as the positive reference standard, and acetate buffer was used as the negative control. (**b**): Antifungal properties of the purified protease inhibitor PDInhibitor. The antifungal properties of PDInhibitor were evaluated against several fungal strains. The fungicidal effect was assessed by measuring the protease inhibitor concentration necessary to kill 50% of the initial inoculum (IC50%), which was deduced from curves obtained from three independent experiments. Cycloheximide was used as the positive reference standard, and acetate buffer was used as the negative control.

Antimicrobial assays of the purified inhibitor from C. *dioscoridis* were carried out separately against bacterial (gram+ and gram− bacteria) and fungal strains. The bactericidal effect was analyzed by measuring the zone inhibition diameter resulting from the anti-proteolytic action of the inhibitor against bacteria. The IC50% values presented in Figure 7a demonstrate that PDInhibitor possesses potent bactericidal effects against *E. coli* (ATCC 25966) and *Bacteroides fragilis* (ATCC 25285), with IC50% values of 13 µg/mL and 13.4 µg/mL, respectively. The tested protease inhibitor was found to be effective against both gram+ and gram− bacteria, and the activity trend was as follows: *E*. *faecalis* (14 µg/mL) > *S. enteric* (14.8 µg/mL) > *K. pneumonia* (17.3 µg/mL) > *B. cereus* (19 µg/mL) and *S*. *epidermidis* (20 µg/mL) strains.

In addition, an antifungal assay of the purified inhibitor was carried out against *A*. *niger, B*. *cinerea*, *F*. *solani* and *P*. *digitatum* strains by measuring the zone of inhibition observed after inoculation of fungi with the purified PDInhibitor in Sabouraud dextrose agar medium and determination of the IC50% values. Figure 7b shows that the highest antifungal effect was observed against *B*. *cinerea,* with an IC50% value of 4.05 µg/mL, and against *A*. *niger* (IC50% 4.7 µg/mL), while the least activity was observed against *P*. *digitatum* (IC50% 6.7 µg/mL) and *F*. *solani* (IC50% 14 µg/mL).

### 2.7. Cytotoxicity of Protease Inhibitors from C. dioscoridis and from Rhamnus Frangula

The cytotoxic effect of the purified PDInhibitor on three different human cell lines (MDA-MB-231, HCT-116, and Lovo) was investigated using MTT assays.

**Figure 8:**
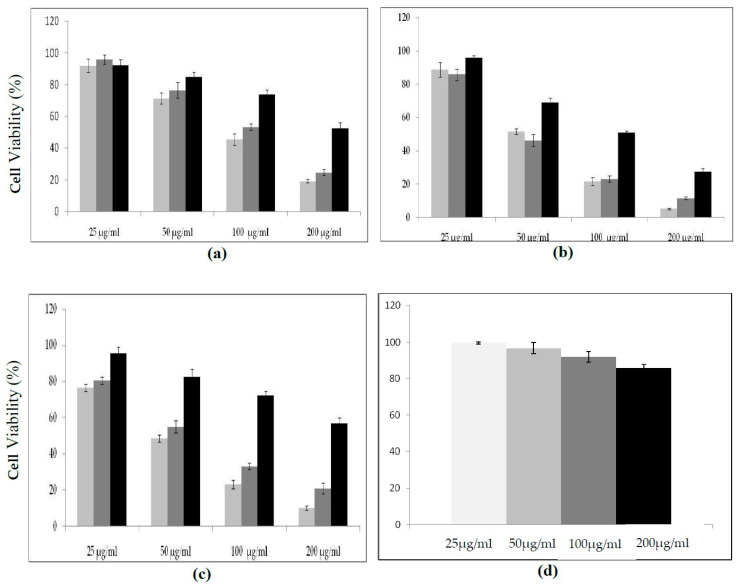
Cytotoxic potency of PDInhibitor in Lovo (**a**), HCT-116 (**b**), and MDA-MB-231 (**c**) cells. Cytotoxicity was assessed using MTT assays by incubating cells for 24 h with various concentrations (25, 50, 100, and 200 μg) of crude inhibitor from *C. dioscoridis* (light gray bars), purified inhibitor *PDInhibitor* (dark gray bars) and pure protease inhibitor from *Rhamnus Frangula* (black bars), (**d**): Cell viability: HUVEC cells were maintained in culture for 72 h with (25–200µg/mL) concentrations of PDInhibitor using MTT solution.

Protease inhibitors at concentrations ranging from 25 to 200 μg/mL were incubated with the appropriate cells in each well for 24 h. When we first measured the cytotoxic effect of protease inhibitors on the human MDA-MB-231 cell line, we noticed that cell viability was strongly affected when using crude or pure inhibitor from *C. dioscoridis* and reached 10.25% and 21%, respectively (at a concentration of 200 µg/mL). However, viability was slightly affected (50%) when the cells were treated with the same amount of pure protease inhibitor from Rhamnus Frangula [24], taken as a control (Figure 8a). Likewise, HCT-116 human cell line viability was affected in the same way and reached 5%, 11% and 27% after pretreatment with crude and pure inhibitor from *C*. *disocoridis* and from *Rhamnus Frangula*, respectively. (Figure 8b). Finally, the percentage of viable Lovo cells in treatment medium containing either protease inhibitor from *C. disocoridis* or from *Rhamnus Frangula* was moderately affected compared to the MDA-MB-231 and HCT-116 cell lines. Indeed, cell viability reached 19%, 24% and 52% after treatment with crude or pure inhibitor from *C. disocoridis* and inhibitor from *Rhamnus Frangula*, respectively (Figure 8c). It is worth noticing that the protease inhibitor did not show any effect on the viability of human umbilical vein endothelial cells (HUVEC) after 72 h at concentrations ranging from 25 to 200 µg/mL, using the MTT (3-(4,5-dimethylthiazol-2- yl)-2,5-diphenyltetrazolium bromide) assay (Figure 8d).

## 3. Discussion

Protease inhibitors are small molecules with molecular masses ranging from 5–25 kDa. [25]. They are found in different living organisms (eukaryotes, prokaryotes, and viruses) [26] and compose a large and diverse family of protease inhibitors [27]. Protease inhibitors act as protective proteins and have aroused great interest in medicine and biotechnology due to their ability to treat immune and inflammation-related diseases, emphysema, arthritis, pancreatitis, and AIDS [28]. Currently, identification of new protease inhibitors from plants is on the rise. Herein, we show extraction, purification, and biochemical characterization of a new protease inhibitor from *C. dioscoridis*. The antimicrobial effect of the inhibitor and its cytotoxicity were also studied.

It is well established that the extraction medium is crucial and improves complete extraction of biomolecules from any desired source. Thus, a variety of solvents were tested for extracting the protease inhibitor from *C. disocoridis*. Extracts prepared with 0.1 M phosphate buffer were much better than those prepared from the other tested solvents because the protease inhibitor activity was maximal (83% ± 3). In fact, several previous studies have shown that 0.1 M phosphate buffer (pH 7.6) allows a high amount of trypsin inhibitor activity from *Cajanus cajan* seeds [29]. After purification of the protease inhibitor, SDS-PAGE analysis of the obtained fractions clearly indicated that the protease inhibitor (named PDInhibitor) possesses a molecular mass of approximately 25 kDa, suggesting that the purified inhibitor protein belongs to the serine protease inhibitor family, characterized by a molecular mass ranging from 18 to 26 kDa [30]. Therefore, a reversed-phase analytical HPLC eurospher 100, C-8 column (250 × 4.6 mm) showed homogeneity of the identified inhibitor. The N-terminal sequence of PDInhibitor showed a high level of identity with those of the Kunitz family [30,31]. In fact, 79% identity was observed with Kunitz trypsin inhibitor A and Kunitz trypsin inhibitor 3, with accession numbers AAF65315.1 and P01070.2, respectively. Moreover, the protease inhibitor was found to be stable in a large pH range, which is an encouraging and interesting characteristic that can be exploited in biotechnological and pharmaceutical industries. The inhibitor was found to be active at pH values ranging from 5.0 to 11.0, with maximal activity at pH 9.0 (87% inhibition). Similar results confirmed that protease inhibitors in the Kunitz family are stable in alkaline pH but lose their full activity at pH values less than 5 [8,32]. In fact, acidic pH extremes seem to affect binding of the inhibitor to the protease. Therefore, some studied inhibitors from the Kunitz family are very sensitive to acidic pH but are stable in the alkaline pH range [32]. Thus, one can conclude that pH affects the activity, structural stability, and solubility of protease inhibitors. Interestingly, PDInhibitor was found to be fully active at 50 °C and maintained 90% of its stability at over 55 °C. Thermal stability is considered an interesting and promising feature for protease inhibitors with potential as therapeutic drugs. Enhancement of the inhibition activity at higher temperatures leads to increases in the efficiency of the inhibitors [33]. Among stabilizer agents, osmolytes (salts, polyols, and amino acids) are known to protect proteins against thermal inactivation [34]. CaCl_2_ (10 mM) was found to be the best stabilizer to support the thermal stability of purified PDInhibitor, followed by BSA (1%). These findings fit well with a previous work showing that, at an elevated temperature of 70 °C, CaCl_2_ (92%) significantly improved thermal stability, followed by glycine (22%) and glycerol (39%). In fact, Ca^2+^ ions improve the thermal stability of protease inhibitors [35]. Most likely, Ca^2+^ ions affect the binding site of the inhibitor with the protease and protect the protein from structural denaturation at high temperature. To better characterize the pure inhibitor for potential use in therapeutic applications, the effect of oxidizing and reducing agents and surfactants on PDInhibitor was examined. All ionic and nonionic surfactants decreased the inhibitory activity except NaTDc and SDS. Indeed, in cell lysis, SDS and protease inhibitors are mixed to maximize the solubilization of biological materials. Remarkably, all the oxidizing agents decreased the protease inhibitor activity. This is possibly due to oxidation of the methionine located in the inhibitor protein reactive site according to Johnson et al. [36]. In fact, it has been reported in a previous work that oxidation of the inhibitor at key catalytic amino acid residues, such as methionine, results in loss of human alpha-1-proteinase inhibitor activity [36]. However, reducing agents (DTT and βME) enhanced the inhibitory activity of PDInhibitor. Bijina et al. [8] suggested that reducing agents affect the functional stability of Kunitz-type protease inhibitors by acting on intramolecular disulfide [30]. Interestingly, when we studied the effect of PDInhibitor on proteases with therapeutic importance, we noticed a remarkably strong affinity with elastase compared to the other proteases tested. In fact, elastase is involved in the pathogenesis of pulmonary emphysema and in inflammatory processes. Thus, the development of inhibitors of low molecular weight, specific for this enzyme and exhibiting good bioavailability, is an active field of academic research and pharmaceutical research. Otherwise, the affinity of purified PDInhibitor with industrially important proteases was much higher with commercially available proteases obtained from *Aspergillus oryzae*, followed by proteases from *Bacillus sp*. These results fit well with previous studies investigating protease inhibitors from *Moringa oleifera* [8]. The biological activities of protease inhibitors are of great interest, such as antibacterial and antifungal effects, especially in the case of pathogenic microorganisms or organisms that are resistant to certain antibiotics. Interestingly, our current results indicated that PDInhibitor was as effective as ampicillin against almost all bacterial strains tested and was particularly potent against *E. coli.* It has been reported that protease inhibitors belonging to the Kunitz family display antimicrobial effects against both gram+ and gram− bacteria, such as *Salmonella typhimurium, Staphylococcus aureus, and Escherichia coli* [37]. In addition, the antifungal effect of the purified protease inhibitor from *C. disocoridis* is attributed to its protective role against phytopathogenic strains [38]. It is well established that proteases are associated with many biological signaling pathways, and several diseases, such as cancer, HIV, infectious diseases, and diabetes, can be treated by inhibiting proteases [13,39]. In fact, protease inhibition has led to treatment of a number of diseases and successful production of many commercial drugs by pharmaceutical companies [40]. In recent years, protease inhibitors have been extensively examined as therapeutic agents, primarily to address various human cancers. Several plant protease inhibitors are under further in vitro evaluation in clinical trials [12]. The current study indicates that *PDInhibitor* presents very low toxicity in normal human cell lines but affects several human cancer cell lines: HCT-116, MDA-MB-231 and Lovo. The viability of MDA-MB-231 breast cancer, HCT-116 colorectal carcinoma and Lovo colon adenocarcinoma cells was significantly inhibited by pure protease inhibitor from *C. dioscoridis* at all concentrations tested (25–200 μg/mL), suggesting pharmacological effects of plant protease inhibitors (PPIs) in cancer prevention. Preclinical (in vitro) studies and pharmacological effects of plant protease inhibitors in disease prevention are well documented. In fact, a protease inhibitor from *Bauhinia bauhinioides* affected the viability of the human prostate cancer cell lines DU145 and PC3 (50–100 μM) [41]. In addition, PIs from *Cicer arietinum* (chickpea) inhibited the viability of MDA-MB-231 breast cancer cells and PC-3 and LNCaP prostate cancer cells (25–400 μg/mL) [42]. However, the current study is a very initial indication that PDInhibitor could have anti-carcinogenic capabilities given its effects on the proliferation of cancer cell lines, but this initial indication might justify more focused and elaborate assays. In addition, the main challenge in the fight against cancer is no doubt finding molecules able to act against pro-carcinogenic proteases and possessing anti-carcinogenic properties.

## 4. Materials and Methods

### 4.1. Materials

Five available proteases with therapeutic importance were used: pure elastase from human leukocytes (Sigma-Aldrich, St. Louis, MO, USA E8140), cathepsin B from bovine spleen (cysteine-peptidase) (Sigma-Aldrich, C6286), α-chymotrypsin (serine peptidase) from bovine pancreas (Sigma-Aldrich, St. Louis, MO, USA C3142), collagenase from *Clostridium histolyticum* (metalloproteinases) (Sigma-Aldrich, St. Louis, MO, USA C2674), and thrombin from bovine plasma (serine protease) (Sigma-Aldrich, St. Louis, MO, USA T7513). Six commercially available proteases were also used: esperase from *Bacillus sp*. (serine-type protease) (Novozyme, Sigma-Aldrich, St. Louis, MO, USA P5860), proteinase K from *Tritirachium album* (serine protease) (Sigma-Aldrich, St. Louis, MO, USA P2308), subtilisin from *Bacillus licheniformis* (*Subtilisin A* is a member of the Serine S8 endoproteinase family) (Sigma-Aldrich, St. Louis, MO, USA P5380), and *Aspergillus oryzae* (fungal protease/peptidase complex produced by submerged fermentation of a selected strain of *Aspergillus oryzae* that contains both endoprotease and exopeptidase activities) (Sigma-Aldrich, St. Louis, MO, USA P6110), *Bacillus licheniformis* (endoprotease of the serine type) (Sigma-Aldrich, St. Louis, MO, USA P4860), and *Bacillus sp*. (a serine-type protease) (Sigma-Aldrich, St. Louis, MO, USA P3111).

### 4.2. Methods

#### 4.2.1. Extraction of Protease Inhibitors

Wild growing *C. disocoridis* plants were collected in Riyadh city in October 2018 (Kingdom of Saudi Arabia), washed, and air dried at room temperature. Then, PI extraction was performed after homogenizing 30 g of the plants in 100 mL of several solvents: 0.1 M phosphate buffer (pH 7.0), distilled water, 15% NaCl (*w*/*v*), 0.05 M HCl, and 0.2% NaOH (*w*/*v*). After 4 h of incubation at room temperature in a rotary shaker at 200 rpm, homogenates were filtered and then centrifuged (12,000 rpm, at 4 °C for 15 min) [29]. The resulting soluble crude extracts were assayed for protease inhibitor activity, and the extract that showed the highest activity was selected for further studies.

#### 4.2.2. Protease Inhibitor Assays

According to the Kunitz method [30] a protease inhibitor assay was performed against trypsin. A 1 mL aliquot at a suitable extract sample dilution was mixed with an equal volume of trypsin (1000 U/mg) and preincubated at 37 °C (15 min). Next, 2 mL of casein (1%) was added. The obtained mixture was incubated for 30 min at 37 °C, and then, 2.5 mL of a 5% trichloroacetic acid (TCA) solution was added to stop the reaction. Finally, the absorbance was measured at 280 nm after centrifugation of the reaction mixture (12,000 rpm, 15 min). One unit of protease inhibitor activity (PIU) was defined as a decrease in absorbance at 280 nm of the TCA soluble casein hydrolysis product, liberated by the action of trypsin per minute under the standard assay conditions [30]. Appropriate controls (reaction without enzyme extract) were run for all assays. Tests were performed in triplicate. The protease inhibitor activity was also expressed as inhibition percentage which was determined by comparison with a control experiment for comparative purposes.

#### 4.2.3. Purification of Protease Inhibitor from C. dioscoridis (PDInhibitor)

Purification of the protease inhibitor was carried out using a combination of ammonium sulfate fractionation (60–90%) precipitation, heat treatment (70 °C, 10 min) and chromatographic filtration using SephadexG-50 gel. The soluble crude extract from *C. dioscoridis* (80 mL, 21,000 PIU) was subjected to ammonium sulfate fractionation (60–90%). The resuspended precipitate obtained after centrifugation at 15,000 rpm for 30 min was subjected to a brief heat treatment at 70 °C for 10 min. The resulting sample exhibiting protease inhibitor activity was loaded on a Sephadex G-50 column (2 × 100 cm) equilibrated with buffer A (0.1 Μ tris HCl buffer, pH 8, containing 0.2 Μ NaCl). The column was eluted at 4 °C with the same solution at a flow rate of 30 mL/hand, and 4.0 mL fractions were collected. The pooled fractions were concentrated using Amicon^®^ stirred cells. The lyophilized PDInhibitor was dissolved in 100 mM phosphate buffer, pH 6.8, containing 150 M NaCl and loaded onto a size exclusion high performance liquid chromatography (HPLC) column (Bio-sil SEC-125, 300 mm × 7.8 mm) previously equilibrated with the same buffer. Elution was performed with the same buffer at 1 mL/min.

#### 4.2.4. Protein Determination

Protein concentration was estimated according to the Bradford method (1976) using bovine serum albumin as a reference. The purified PI was analyzed via 15% sodium dodecyl sulfate polyacrylamide gel electrophoresis (SDS-PAGE) as described by Laemmli [43].

#### 4.2.5. Amino Acid Sequencing

The N-terminal sequence of pure PDInhibitor was determined by automated Edman’s degradation using an Applied Biosystems Protein Sequencer Procise 492 equipped with a 140C HPLC system. The N-terminal sequencing was done by Prf. Hafedh mejdoub, sequencing unit life science department, FSS - Faculté des Sciences de Sfax. Sfax University.

#### 4.2.6. Effect of pH and Temperature on PDInhibitor Activity and Stability

Buffers at pH values ranging from 3 to 13 were used to test the protease inhibitor activity at 37 °C. One percent of the casein substrate was prepared using the following buffers at 200 mM: sodium acetate buffer (pH 3–5), potassium phosphate buffer (pH 6–7), Tris–HCl buffer (pH 8–9), and glycine-NaOH buffer (pH 10–13). The pH stability of the PI was determined by incubating the protease (12 h at 4 °C) at various pH values ranging from 2 to 13. The residual protease inhibitor activity was determined under the standard assay method after centrifugation (12,000 rpm, 30 min). Measurements were performed in triplicate. In addition, the effect of temperature on protease inhibitor activity was determined by testing the enzyme activity at temperatures ranging from 20 to 70 °C at pH 7. Finally, to assess thermal stability, the protease inhibitor was incubated at temperatures ranging from 30–90 °C at pH 7 for 1 h. Residual activity was measured after centrifugation (30 min at 12,000 rpm) under the standard assay method.

#### 4.2.7. Influence of Stabilizers on PDInhibitor Thermostability

The thermostability improvement of the purified PI at 80 °C was evaluated by investigating the effect of addition of several known thermal stabilizers: 1% BSA, 10 mM CaCl_2_, 10 mM cysteine hydrochloride, 10% glycerol, 1 M glycine, 10 mM PEG 8000, 10% sorbitol, 1% casein, 1% starch, 1% sucrose, and 10 mM urea. Protease inhibitor activity was measured after 2 h of incubation at 80 °C.

#### 4.2.8. Influence of Metal Ions on PDInhibitor Activity

Divalent ions (such as Ca^2+^, Cd^2+^, Co^2+^, Fe^2+^, Hg^2+^, Mg^2+^, Mn^2+^, and Zn^2+^) were added to the reaction mixture at final concentrations of 1 and 10 mM. The influence of metal ions on protease inhibitor activity was tested after incubation at 37 °C for 1 h. Assays were performed under optimal conditions.

#### 4.2.9. Effect of Reducing/Oxidizing Agents and Surfactants on PDInhibitor Activity

The effect of both reducing agents (β-mercaptoethanol (βME) and dithiothreitol (DTT) at concentrations ranging from 0.2 to 1% (*v*/*v*)) and oxidizing agents (hydrogen peroxide (H_2_O_2_), dimethyl sulfoxide (DMSO) and sodium hypochlorite (NaOCl) at concentrations ranging from 1–5% (*v*/*v*)) on *PDInhibitor* activity was tested by incubating the appropriate agent with the protease inhibitor for 30 min. Residual activity was then measured. Moreover, in the same manner, the effect of nonionic and ionic surfactants (NaTDC, SDS, Triton X-100, Tween-80, and Tween-20) on *PDInhibitor* activity was tested by incubating the protease inhibitor with surfactant for 60 min. Residual activity was estimated in the mixture dialyzed against 0. 1 M phosphate buffer (pH 7).

#### 4.2.10. Effect of PDInhibitor on Available Important Therapeutic and Commercial Proteases

The activities of five available proteases with therapeutic importance were determined according to previously reported protocols [44,45,46]. The enzyme inhibition using *PDInhibitor* (0.25 mg/mL) was determined after preincubation for 15 min, and then, the remaining enzyme activity was measured. PDInhibitor activity on the respective enzyme is expressed as percent inhibition. Then, the effect of the studied protease inhibitor on six available commercial proteases was measured in the same manner: the inhibitor (0.25 mg/mL) was incubated with the respective reaction mixture for 10 min. The residual enzyme activity is expressed as the inhibition percentage.

#### 4.2.11. Antimicrobial Activity of PDInhibitor

The antibacterial and antifungal activities of the purified protease inhibitor were evaluated against the following gram-positive strains: *Bacillus cereus* (ATCC 14579), *Bacillus subtilis* (ATCC 6633), *Enterococcus faecalis* (ATCC 29122), *Staphylococcus aureus* (ATCC 25923), *Staphylococcus* *epidermidis* (ATCC 14990); gram negative strains: *Salmonella* enteric (ATCC 43972), *Pseudomonas aeruginosa* (ATCC 27853), *Klebsiella pneumonia* (ATCC 700603), and *Escherichia coli* (ATCC 25966); and fungal strains: *Aspergillus niger, Botrytis cinerea, Fusarium solani and Penicillium digitatum*. Bacterial viability was assessed by measuring the colony forming ability (CFU) of bacteria incubated in the absence or presence of the purified *PDInhibitor.* Initial mixtures containing both 2 × 10^7^ CFU/mL in sterile brain heart infusion (BHI) and the purified *PDInhibitor* at the appropriate amount were incubated for 2 h under shaking at 37 °C. Moreover, to calculate bacterial viability, we performed a serial dilution into the culture medium before spreading colonies onto agar plates. Based on colony counting after incubation for 24 h and at 37 °C, the bactericidal effect of the purified *PDInhibitor* is expressed as the residual number of CFU. Thus, we calculated IC50% values (or half-maximal (50%) inhibitory concentrations) that correspond to the amount of protease inhibitor capable of killing 50% of the starting inoculums. The antifungal potency of the pure *PDInhibitor* was evaluated using the well diffusion method with Sabouraud dextrose agar. The protease inhibitor was placed in sterile paper discs deposited onto the center of the inoculated Petri dishes, and then, the dishes were incubated at 30 °C for 24 h. For further comparison, a positive control, cycloheximide (1 mg/mL), was used under the same conditions.

#### 4.2.12. Cytotoxicity of Protease Inhibitors

Cell viability was investigated in HUVEC, HCT-116, Lovo, and MDA-MB-231 cells using different amounts of both crude and purified protease inhibitor from *C. dioscoridis.* Pure protease inhibitor from *Rhamnus Frangula* was also studied as a control. The cytotoxicity assay was performed using MTT (3-(4,5-dimethylthiazol-2-yl)-2,5-diphenyltetrazolium bromide) assay, first described by Tim Mosmann in 1983 [47]. This colorimetric assay uses reduction of a yellow tetrazolium salt MTT to measure cellular metabolic activity as a proxy for cell viability. Viable cells contain NAD(P)H-dependent oxidoreductase enzymes which reduce the MTT reagent to formazan, an insoluble crystalline product with a deep purple color. Formazan crystals are then dissolved using a solubilizing solution and absorbance is measured at 500–600 nanometers using a plate-reader. Briefly, 4 × 10^4^ cells (in each well) were incubated in 96-well plates at 37 °C for 24 h with various amounts of protease inhibitor samples diluted in culture medium. Addition of 20 μL of freshly prepared MTT (5 mg/mL in PBS) to the cells was followed by incubation at 37 °C for 4 h in a 5% CO_2_ incubator. A volume of 180 μL of saline solution (50:50) was added to an equal volume of the preparation medium. To dissolve the formazan crystals formed, homogenization was performed using a plate shaker. The absorbance was measured at 550 nm using a micro plate reader. The cell viability results are expressed as a percentage of the OD values at 500 mm for protease inhibitor-treated cells relative to the control.

## 5. Conclusions

*Conyza. dioscoridis* is a source of bioactive compounds with many virtues. In the current study, the biological effects of protease inhibitors from *C. dioscoridis* are reported. The purified inhibitor was biochemically characterized as pH and temperature stable, and the effect of oxidizing and reducing agents and stabilizers was investigated. Our results indicate that the purified PDInhibitor is a potential protease inhibitor candidate with therapeutic importance in pharmaceutical industry applications due to its antibacterial, antifungal, and cytotoxic potential. Thus, *C. disocoridis* is an efficient source of protease inhibitors with high potential in biotechnological applications.

## Figures and Tables

**Table 1:** Activity of protease inhibitor extracted from C. dioscoridis using different solvents.

Extraction Solvent	Protease Inhibitor Activity (%)
Phosphate buffer 0.1 M	83.3 ± 3.05
Distilled water	47.3 ± 2.5
NaCl 15%	66,3 ± 4.1
HCl 0.05 M	35 ± 3.6
NaOH 0.2%	18.6 ± 3.5

**Table 2:** Flow sheet of PDInhibitor purification.

Purification Step	Total ^a^ Activity (units)	Protein ^b^ (mg)	Specific Activity (PIU/mg)	Activity Recovery (%)	Purification Factor
Extraction	21,000	1350	15.5	100	1
Ammonium sulfate fractionation(60–90%)	18,100	520	34.8	86.2	2.2
Heat treatment (70 °C, 10 min)	12,350	56	220.5	58.8	14.2
Sephadex G-50	5470	4.6	1189.1	26	76.7

^a^ 1 Unit: One unit of protease inhibitor activity was defined as a decrease by one unit in absorbance of TCA soluble casein hydrolysis product liberated by trypsin action at 280 nm per minute under the assay conditions. ^b^ Proteins were estimated using the Bradford method [21]. The experiments were conducted three times.

**Table 3:** Effects of surfactants and oxidizing/reducing agents on the stability of PDInhibitor activity.

Detergents		
Surfactant	Concentration (%)	Residual Activity (%)
**Tween-20**	1	80 ± 4.2
**Tween-80**	1	64.5 ± 3.5
**Triton-X100**	1	63.9 ± 4.1
**SDS**	1	141 ± 8.4
**NaTDC**	1	193.5 ± 6.3
**Oxidizing agents**		
**H_2_O_2_**	1	58.35 ± 3.3
	2	40.75 ± 3.1
	3	26.15 ± 3.3
	4	18.45 ± 2.1
	5	8.1 ± 1.2
**NaOCl**	1	64 ± 4.2
	2	37.2 ± 3.1
	3	21.2 ± 2.2
	4	10.15 ± 1.6
	5	6.05 ± 0.9
**DMSO**	1	89.7 ± 3.8
	2	83.15 ± 3
	3	74.6 ± 3.6
	4	64.9 ± 2.6
	5	50.5 ± 3.5
**Reducing agents**		
**DTT**	0.2	107 ± 4.2
	0.4	133 ± 5.6
	0.6	144 ± 4.2
	0.8	157.5 ± 3.5
	1	160 ± 4.2
**βME**	0.2	103 ± 2.8
	0.4	122 ± 4.2
	0.6	139.5 ± 3.5
	0.8	151.5 ± 3.5
	1	167 ± 2.8

The results are the relative protease inhibitor activity expressed as a percentage of the maximum activity recorded without the addition of compound. Data are the mean ± SD (*n* = 3).
